# Orbital Venolymphatic Malformation Treated With Sodium Tetradecyl Sulfate: A Case Report

**DOI:** 10.7759/cureus.29173

**Published:** 2022-09-14

**Authors:** Sucharita Das, Ajai Agrawal, Sandeep K Burathoki, Khanak K Nandolia, Aarzoo Juneja, Ramanuj Samanta

**Affiliations:** 1 Ophthalmology, All India Institute of Medical Sciences, Rishikesh, Rishikesh, IND; 2 Intervention Neuroradiology, All India Institute of Medical Sciences, Rishikesh, Rishikesh, IND; 3 Radiology, All India Institute of Medical Sciences, Rishikesh, Rishikesh, IND

**Keywords:** digital subtraction angiography, dural arterio-venous fistula, sodium tetradecyl sulfate, sclerotherapy, venolymphatic malformation

## Abstract

Orbital and periorbital venolymphatic malformations (VLMs) are benign congenital vascular lesions and constitute 1%-3% of all orbital masses. Widespread facial venous malformations have a high incidence of associated intracranial developmental venous anomalies (DVAs). In such cases, there can be a sudden increase in proptosis following upper respiratory infection or minor trauma. Numerous percutaneous intralesional sclerosing agents like sodium tetradecyl sulfate (STS), bleomycin, doxycycline, ethanol, and OK-432 (Picibanil) have been used for treating VLMs. We hereby report a rare case of retro-orbital VLM treated successfully with STS injection and an isolated dural arterio-venous (AV) fistula in the same patient.

## Introduction

Orbital and periorbital venolymphatic malformations (VLMs) are benign congenital vascular lesions and constitute 1%-3% of all orbital masses [1]. Complications include intralesional hemorrhage, cellulitis, amblyopia, blepharoptosis, periocular edema, proptosis and impaired visual acuity (VA) [2]. It is established that patients with widespread facial venous malformations have a high incidence of associated intracranial developmental venous anomalies (DVAs) [3]. Various percutaneous intralesional sclerosing agents like sodium tetradecyl sulfate (STS), bleomycin, doxycycline, ethanol and OK-432 (Picibanil) have been used as first-line treatment for VLMs elsewhere in the body [4]. We describe a rare case of retro-orbital VLM treated successfully with STS injection and an isolated dural arterio-venous (AV) fistula in the same patient.

## Case presentation

**Figure 1:**
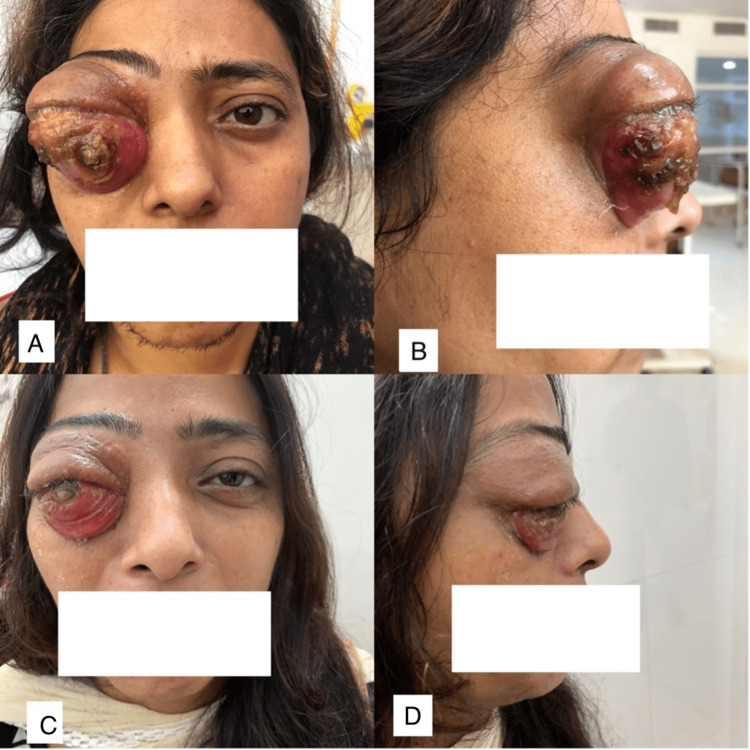
Clinical picture of patient A and B, Front and lateral view of patient (pretreatment). C and D, Front and lateral view of patient (post-treatment by STS injection. STS: sodium tetradecyl sulfate.

A 30-year-old female presented with protrusion of the right eyeball for 1 ½ years and diminution of vision in the right eye for one year as depicted in Appendix 1, Figures 1A, 1B, respectively. She had downward displacement of the right eye with drooping of the right upper eyelid since birth. In 2011 (around 10 years back), she underwent surgery in the right orbit (the nature of the surgery is not known from available documents), following which the swelling subsided. She also underwent surgery for excision of AV malformation of the right cheek with excision of soft tissue on the right upper eyelid, under general anesthesia, in 2015. In 2020, she had a fall from a two-wheeler, following which she developed mild protrusion of the right eye which increased significantly in the last two months. It was progressive, associated with a diminution of vision and dull boring pain. There was no variation with posture or strain, no double vision, fever or tinnitus. Diminution of vision in the right eye was gradual, painful and progressive in nature. Examination revealed a perception of light in the right eye with an inaccurate projection of rays in all quadrants. The patient exhibited marked abaxial proptosis, conjunctival chemosis, epithelial keratinization and old scar medially in the upper eyelid without distortion of lid contour. On palpation of the lesion, its consistency was soft, tender and non-compressible. No thrill/pulsation was felt, the size of the lesion did not increase with the Valsalva maneuver, and there were no palpable lymph nodes. Fundus could not be visualized due to extensive keratinization of the cornea. Extraocular movements were restricted in all gazes. Ophthalmic examination of the left eye was unremarkable with an uncorrected visual acuity (UCVA) of 20/20.

**Figure 2:**
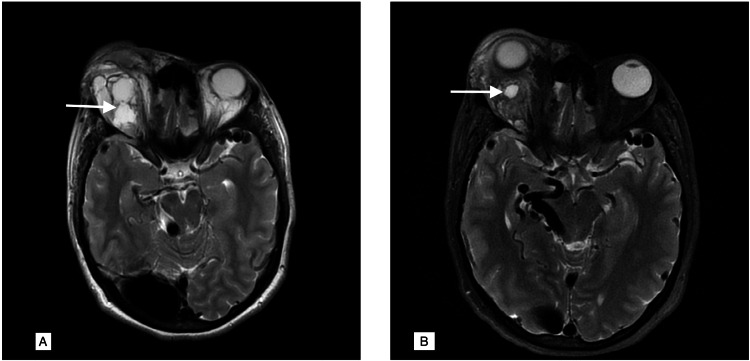
Orbit and brain magnetic resonance imaging (MRI) A. Axial T2-weighted imaging showed large lobulated T2 hyperintense retro-orbital cystic lesion (white arrow). Thin septations are seen within the cystic lesion. B, Axial T2-weighted imaging at three-month follow-up showed significant reduction in volume of the retro-orbital lesion and altered signals reminiscent of thrombosis and/or fibrosis (white arrow).

Orbit and brain magnetic resonance imaging (MRI) revealed proptosis of the right eyeball. There was a large heterogeneous signal intensity, lobulated cystic mass of 37×28×31 mm with soft tissue component, epicentered in lateral extraconal and intraconal compartments of the right orbit. The mass was displacing the lateral rectus and optic nerve medially and right globe anteriorly with edematous thickening of both upper and lower eyelids. No areas of diffusion restriction/blooming were seen. Loculi of the orbital lesion showed differential signal intensity fluid within the lesion as shown in Appendix 2, Figures 2A, 2B. There were two discrete multicystic lobulated lesions involving the right orbit, right masticator and right infratemporal space (probably slow-flow vascular malformations of venous/lymphovenous origin). Dural AV fistula with retrograde reflux in the superficial and deep venous system was also seen.

**Figure 3:**
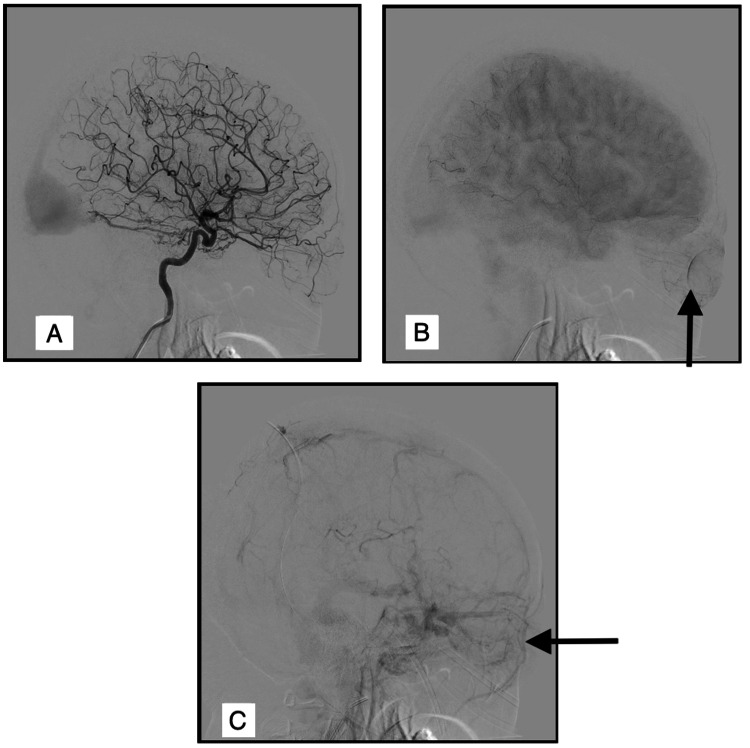
Digital subtraction angiography images A, Selective right internal carotid artery injection showed mild stretching and displacement of orbital branches of ophthalmic artery. B, Choroid blush is preserved (black arrow). C, Venous phase showed congested ophthalmic veins (black arrow).

On digital subtraction angiography (DSA), no abnormal vascular blush was seen in the right retro-orbital mass lesion in the arterial or venous phase, confirming veno-lymphatic nature of the lesion. Choroidal blush was also preserved as depicted in Appendix 3, Figures 3A-3C. Incidentally, a high-flow dural AV fistula was seen at the right transverse sinus, feeding from a transosseous branch of the right occipital artery and a petrosal branch of the right middle meningeal artery. There were no connections of the dural AV fistula with the right retro-orbital lesion. The patient was diagnosed as a case of retro-orbital VLM and an isolated high-flow dural AV fistula and planned for injection of STS for the orbital lesion.

Procedure for sclerotherapy

Under conscious sedation and ultrasonic guidance, the lateral portion of the retro-orbital mass lesion was punctured and approximately 5 ml of altered chocolate-colored fluid was aspirated from the lesion; then 3 ml of contrast was injected under fluoroscopy to confirm needle position and to rule out any communication with the cavernous sinus. Contrast stagnation was seen with a fluid-fluid level at the dependent position. Then approximately 3 ml of STS was injected into the lateral loculation of the lesion of the orbit under ultrasonic guidance. The patient tolerated the procedure well, although there was a mild increase in soft tissue swelling after the procedure.

At one day post-sclerotherapy, she had pain and moderate, localized inflammatory reaction, including eyelid edema, ecchymosis and chemosis which were managed with morphine in a dose of 10 mg four hourly. There were no systemic complications. The patient was followed up at regular intervals, and at three months post-sclerotherapy, there was a remarkable decrease in proptosis of her right eye as shown in Appendix 1, Figures 1C, 1D. The post-procedure visual acuity is the perception of light in the right eye with the inaccurate projection of rays in all quadrants and 20/20 in the left eye. She is currently under our follow-up and has no fresh complaints. Since the patient is presently asymptomatic with respect to the dural AV fistula, no intervention was planned for the same.

## Discussion

Other than VLMs, the differential diagnosis of retro-orbital lesions causing proptosis includes inflammatory, traumatic and neoplastic conditions. VLMs are rare, benign congenital lesions that are generally treated by interventional techniques similar to other slow-flow vascular malformations. Periorbital VLMs are known to coexist with discrete intracranial vascular anomalies including developmental venous anomalies, dural AV malformations, occlusion or absence of dural sinuses and jugular veins, cerebral cavernous malformations and pial AV malformations, as was seen in our patient who had a dural AV fistula. Hence this correlation emphasizes the relevance of ruling out intracranial vascular malformations, which are generally asymptomatic and incidentally detected but in some cases may present with hemorrhage, headaches or other neurological symptoms. Due to this, contrast-enhanced MRI of the brain and orbits is advocated upon diagnosis of lymphatic malformations [3,5].

It is challenging to treat orbital VLMs. They are irregular, infiltrative lesions involving adjoining vital structures of the orbit. They can bleed torrentially due to abundant vascular beds. For lesions that do not threaten vision or cause cosmetic issues, conservative management is suggested [6]. Various techniques for managing VLMs have been described such as surgery, sclerotherapy, laser therapy, cryotherapy and electrocoagulation treatment [7].

We chose intralesional sclerotherapy as the treatment modality in this case. Sclerotherapy is defined as the introduction of a foreign substance into the lumen of a vessel, causing thrombosis and subsequent fibrosis [6]. In our case, 3 ml of STS was injected in the superolateral quadrant of the right orbit under ultrasonic guidance. STS is a clear, colorless, surface-active substance composed of sodium 1-isobutyl-4-ethyloctyl sulfate plus 2% benzyl alcohol; it is supplied in 2-ml ampoules of 1% and 3% solution. It is increasingly being used as a safe and potent solution for sclerosing undesirable veins [6].

STS acts by causing endothelial necrosis via direct cytotoxic action. This agent was chosen due to its relative high potency compared to various other available agents. However, its disadvantage is it produces remarkable swelling in the first one to five post-procedure days, injection site pain, skin eruptions, skin discoloration, superficial thrombophlebitis and grave adverse effects such as anaphylactic shock and possible neurological injury [2,4]. Microcystic lesions (< 10 mm) can be managed by doxycycline, sirolimus or surgery, whereas macrocystic lesions can be treated by the stimulation of the lining endothelium by STS, bleomycin, Picibanil (OK‑432) or 60%-100% ethanol [8,9].

Proptosis decreased significantly in our patient two to three months post-sclerotherapy. We are planning a second sitting of STS injection depending on the patient’s willingness for the procedure. In a previous report by Barnacle et al. post-STS injection in 29 patients, 18 had improved visual acuity and three had stable vision. A full radiological remission of the malformation was seen in 15 patients (51.7%), and a good response was shown in 11 patients (37.9%) [2]. However, there are very few reports of the management of orbital VLM by STS in the literature.

## Conclusions

Ultrasound-guided STS injection for orbital VLMs is a repeatable and safe technique in expert hands, and the subsequent radiological result is remarkable. Management by a multidisciplinary team including a specialist interventional neuroradiologist and an experienced ophthalmologist is imperative. Sclerotherapy is strongly recommended as the first-line treatment of orbital VLMs. We also emphasize the need for ruling out intracranial vascular malformations, in patients with orbital VLMs, which are generally asymptomatic in some cases but may present with hemorrhage, headaches or other neurological symptoms.

## Appendices

Appendix 1 

Appendix 2 

Appendix 3 
